# The Pvc15 ATPase selectively associates effector proteins with the *Photorhabdus* virulence cassette

**DOI:** 10.1098/rsos.240948

**Published:** 2024-10-23

**Authors:** Rhys Evans, Nicholas R. Waterfield

**Affiliations:** ^1^ Warwick Medical School, University of Warwick, Coventry CV4 7AL, UK

**Keywords:** virulence factor, eCIS, toxin delivery, bacteria, ATPase, effector

## Abstract

The *Photorhabdus* virulence cassette (PVC) is an extracellular contractile injection system. In the producing bacterium, N-terminal signal peptides enable effector ‘payloads’ to be loaded into the PVC’s hollow tube—facilitated by the ‘ATPases associated with diverse cellular activities’ (AAA) ATPase, Pvc15—ready for injection of the toxin or virulence factor into eukaryotic cytosols. Pvc15’s function and its interaction with the signal peptide were unclear. This study describes the signal peptide diversity in extracellular contractile injection system clades and interrogates the Pvc15–signal peptide interaction using ATPase assays, cell respiratory assays and western blot quantification of *Escherichia coli* lysates and co-purifications of PVCs with their payloads. This study found that extracellular contractile injection system signal peptides can be grouped according to sequence alignment, owing to potentially homologous loading mechanisms. Pvc15 contains three domains, including tandem AAA domains D1 and D2. By constructing Pvc15 mutants, we found that while each domain is necessary for PVC-payload loading, domain D2 is the sole bioactive ATPase domain and rescues unstable payloads via the signal peptide. Finally, truncating the signal peptide abolishes Pvc15-dependent PVC loading and has varying effects on payload stability. This study provides crucial insights into extracellular contractile injection system effector loading mechanisms and their ATPase chaperones, and suggests that these devices could be bioengineered for injection of therapeutic proteins into human cells.

## 1. Introduction

The *Photorhabdus* virulence cassette (PVC) is an example of a class of bacterial bioactive protein delivery devices collectively termed extracellular contractile injection systems (eCIS) [1–3]. Devices of this nature are often large (>10 MDa) multiprotein phage-like nanosyringes consisting of a rigid, hollow tube whose distal spike is used to puncture the cell membrane of the target host cell after contraction of the surrounding interlinked sheath proteins to deliver a toxic effector ‘cargo’ or ‘payload’ [4–6]. *Photorhabdus* species encode 5 or 6 PVC operons in their genomes, each differing only slightly in the conservation of the PVC core structural components [1]. Instead, operons differ in their effector cargoes, which are encoded downstream and which give name to the operon itself, such as Pnf, Pdp1, Cif, LopT, PaTox and unit 4 [7,8]. Cargo loading is mediated, in part, by the presence of 50–70 amino acid N-terminal disordered ‘signal peptides’ (SPs), which are necessary to load many copies of payload into the PVC tube lumen [8,9]. The SPs can be fused to heterologous proteins for loading, such as β-lactamase, monomeric red fluorescent protein (mRFP), *Renilla* luciferase, the cancer cell-killing *Trichosanthes kirilowii* trichosanthin, and Cre recombinase [8–11].

Heterologous loading and injection of payloads offer an exciting potential for PVCs to be bioengineered for the specific delivery of therapeutic proteins into the cytosol [12]; Pvc13 tail fibres can be engineered with epitope tags or adenovirus binding domains to retarget PVCs to cell types [10].

How SPs target payloads to the PVC has remained largely elusive, though an interaction between Pvc15 and the SP was previously shown to be required for PVC loading and unfolding of SP-associated mRFP [9]. PVCs can assemble without Pvc15 but lack an associated payload [13], implicating its role in payload loading. Since SPs are highly disordered polypeptides [10], it was hypothesized that the SP may confer instability to its payload via bacterial protein degradation machinery.

To understand the relevance of SPs to PVCs and their homologues, this study presents an analysis of eCIS putative effector diversity in the minor eCIS clade consisting exclusively of Gram-negative bacteria: lineage I. Protein structure prediction was used to identify the ubiquity of SPs in lineage Ia: the sub-clade encompassing the PVC and its structural homologues, including the anti-feeding prophage (Afp) from *Serratia* and *Yersinia* [3]. The structures of Pvc15 monomers and hexamers were predicted to identify key residues, motifs and domains for informing experimental analyses. Western blot was used to investigate how SPs from PVC effectors, Pnf and Cif, influence Pvc15 interactions and payload stability when expressed in *Escherichia coli*. The effect of SP length on payload stability and loading was investigated by truncating the 50 amino acid Pnf and Cif SPs (Pnf50 and Cif50) to 10 amino acids (Pnf10 and Cif10). Finally, Pvc15 mutants were constructed to analyse its ATPase activity and identify relevant domains and residues for its role in loading cargo into the PVC.

## 2. Material and methods

### 2.1. Plasmid constructs

The pVTRa vector [14] was used for payload expression by the addition of 1 mM isopropyl β-d-1-thiogalactopyranoside (IPTG). In this construct, an N-terminal SP, such as Pnf50, is encoded followed by a short ‘VD’ linker. The main cargo protein in this study was the Pnf effector, residues 51–340. Finally, a short ‘LQ’ linker was encoded before a Myc tag to visualize payload by western blot. The pBAD-PVC*pnf* vector was constructed by synthesizing the PVC*pnf* operon encoding FLAG (DYKDDDK) to the C-terminus of Pvc16 and was induced with 0.2% arabinose [15] in BL21(DE3) *E. coli*. An epitope tag on one of the PVC*pnf* proteins was also used for immunoprecipitation of mature PVC*pnf*.

The pVTRaDuet vector, which encodes *pvc15* and *pnf* under the control of the same promoter, was generated by PCR of *pvc15* with overhangs encoding hexahistidine tags and the relevant restriction enzyme sites into pVTRa: *Kpn*I and *Bam*HI using primers: forward 5′-GGTACCTTGACCACAATGACTTAGTCTGAGTAAAAAATATGCACCACCACCACCACCACAATATATCGCCTGTTTTTTATGATTCATTG-3′; reverse 5′-GGATCCTCCTCAATGATGATGATGATGATGAAATGTTAATCGTCCGACTTTAGC-3′. In addition, the pBAD-PVC*pnf*Δ*pvc15* vector was made by overlap extension PCR and restriction ligation of *pvc14* and *pvc16* fragments using overlap/inside primers: forward 5′-CGTGGTTATAACCATTGACCACAGAGAGGTTTTTTATGTTAAACACGCAAAC-3′; reverse 5′-GTTTGCGTGTTTAACATAAAAAACCTCTCTGTGGTCAATGGTTATAACCACG-3′, and outside primers for subsequent restriction ligation using *Pst*I and *Sal*I: forward 5′-GTCTGCAGACGCACGATC-3′; reverse 5′-GATGATGATGATGATGGTCGACG-3′. Restriction sites are underlined.

Truncations of the Pnf50 and Cif50 SPs (Pnf10 and Cif10, respectively) were conducted by overlap extension PCR and restriction ligation of pVTRa fragments containing *Bss*HII and *Sal*I sites using primers Pnf10: forward 5′-gcaatGCGCGCcattacc-3′; reverse 5′-tttGTCGACtacggtctgaggattagc-3′; and Cif10: reverse 5′-tttGTCGACgcaatcatcttctttactg-3′). Similarly, the E555Q point mutation to Pvc15 in the pVTRaDuet vector could be made using overlap extension PCR and restriction ligation using primers: forward 5′-ggtattattctttgatCaagctgacgcactg-3′; reverse 5′-cagtgcgtcagcttGatcaaagaataatacc-3′; domain deletions were done by introducing the *Kpn*I or *Bam*HI sites into the C-terminal domain fragment or N-terminal domain fragment, respectively, using primers: forward 5′-atgggtaccatgcaaaatttcggtcaattggcaca-3′; reverse: 5′-agcggatccTCCtcataccggtatctttttcagccatacc-3′.

### 2.2. Protein expression

Transformed BL21(DE3) *E. coli* (New England Biolabs, NiCo21;) were incubated overnight in a starter culture of 10 ml at 180 r.p.m. and 37°C with the appropriate antibiotic(s); each biological replicate was grown into a separate starter culture. Overnight cultures were diluted 1 : 100 and grown again for 3–4 h to OD_600_ = 1.0. 0.2% arabinose was added to induce expression of the pBAD-PVC*pnf* vector and 1 mM IPTG was added to induce the pVTRa vector encoding the cargo. Cells were then incubated at 200 rpm and 25°C.

For measuring cargo stability, aliquots were taken 3 h post-induction. For measuring degradation of the cargo, kanamycin was added at a final concentration of 20 μg ml^−1^ and aliquots were taken after 4 h. For PVC purification, cells were incubated overnight after induction and then centrifuged at 3000*g* for 20 min at 4°C. Cells were then lysed using 10 ml of lysis buffer per gram of pellet, prepared as follows: 200 μg ml^−1^ lysozyme, 0.5% (v/v) Triton X-100, 1 tablet complete EDTA-free protease inhibitor per 50 ml, and 70 μl per 100 ml DNase I. After resuspension, cells were homogenized using a cell homogenizer at 30 kpsi (One Shot Model; Constant Systems).

At each stage of purification, 25 μl of sample was taken and added to 25 μl 2× lithium dodecyl sulfate buffer with 50 mM DTT and heated for 20 min at 95°C. The homogenized sample was then spun at 12 000*g* for 35 min at 4°C and the supernatant was filtered through a syringe filter with 0.45 μm pore size.

PVC*pnf* was then purified by immunoprecipitation of a PVC*pnf* structural protein via an epitope tag; mature nanosyringes were verified using transmission electron microscopy (TEM).

### 2.3. Immunoblotting and quantification

SDS-PAGE was conducted using 1 : 1000 rabbit anti-FLAG (DYKDDDDK, Cell Signaling, 14793) or mouse anti-Myc (Cell Signaling, 2276) monoclonal antibodies and visualized using 1 : 20 horseradish peroxidase (HRP)-bound secondary antibody with luminol.

For quantification of band intensities, samples were normalized for each band by a corresponding loading control in the form of either a fixed-point band which appeared in all samples, such as the FLAG-tagged Pvc16 cap protein for PVC loading gels, or β-RNA polymerase (β-RNAP) for comparisons of target proteins in cell lysates. For measurements of Pnf abundance from cell lysates, quantified bands were normalized staining the polyvinylidene fluoride (PVDF) membrane with Coomassie blue stain then summing all points in each corresponding lane using FIJI (ImageJ); this method is ideal for reducing the variance in normalized data [16–18].

### 2.4. Resazurin assay for cell respiration

Raji cells grown in RPMI-1640 medium (Gibco) with 2 mM l-glutamine, 10% fetal bovine serum (FBS) (Gibco), 100 units ml^−1^ penicillin and 100 µg ml^−1^ streptomycin incubated under 5% CO_2_ at 37°C for 2 days were diluted to 8 × 10^5^ cells ml^−1^. Cells were seeded into 100 μl aliquots in quadruplicate in a black, clear-bottomed 96-well plate. The plate was incubated for 1 h under 5% CO_2_ at 37°C. Phosphate-buffered saline (PBS) or 10 μg of PVC sample was added and incubated for a further 4 h. Finally, resazurin was added to each well in quick succession to a final concentration of 5 μM. The plate was incubated for a further 6 h and fluorescence was measured: excitation = 530–570 nm, emission = 580–620 nm.

### 2.5. ATPase activity

The colorimetric ATPase assay kit (Abcam, ab234055) was used. 15 ml of *E. coli* BL21(DE3) cultures were grown to OD_600_ = 1.0 and induced with 0.2% arabinose and 1 mM IPTG to co-express PVC*pnf*Δ*pvc15* and the SP-associated payload with or without Pvc15 for 16 h then processed according to the manufacturer’s instructions.

### 2.6. Statistical testing and data visualization

While a two-way ANOVA with planned comparisons via estimated marginal means would have been suitable in most cases since some all-versus-all comparisons would be invalid (for example, WT SP with Pvc15 vs truncated SP without Pvc15), given the small sample sizes, it was preferred to use Student’s *t*-tests [19] and then adjust the *p*-values for multiple comparisons using the Holm–Bonferroni method [20]. Error bars were made to represent standard deviation when showing data variability, or standard error of the mean when it was desirable to show the precision of the attained mean, such as in the ATPase assay.

All charts and graphs were made using R in RStudio via the ggplot2 package [21–23].

### 2.7. Protein structure prediction

All protein structures shown were generated using either AlphaFold 2.0 via the ColabFold suite [24,25], or using MoLPC for large multi-protein structure predictions [26].

## 3. Results

### 3.1. Extracellular contractile injection system lineage I analysis reveals diverse putative effectors

**Figure 1.**
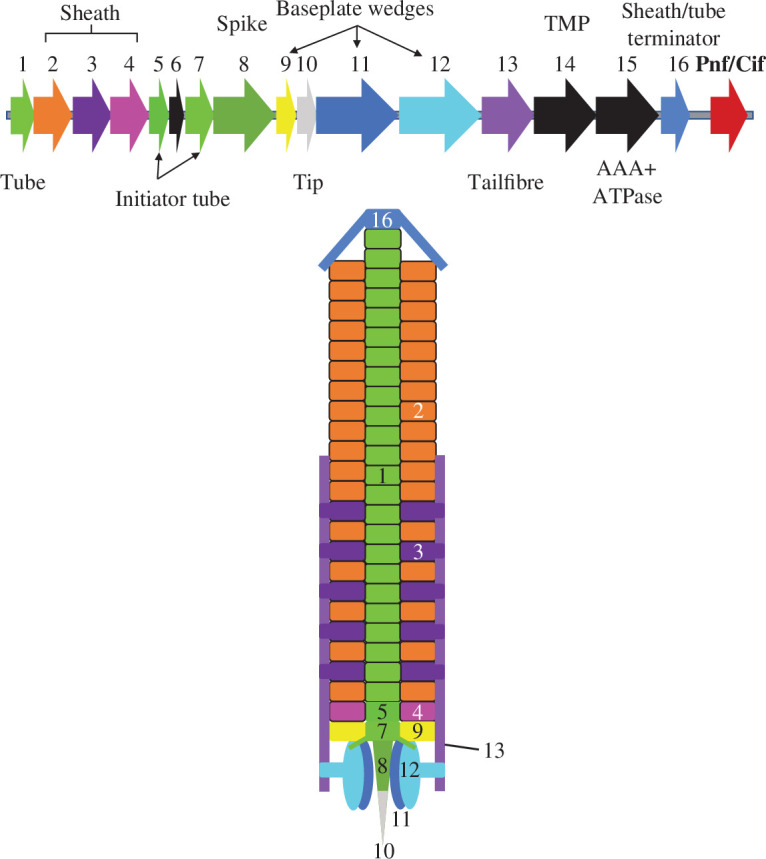
*Photorhabdus* virulence cassettes and their extracellular contractile injection system (eCIS) lineage I homologues consist of a 16-gene operon of structural and accessory components followed by downstream open reading frames (ORFs) encoding putative payload effectors. A labelled cartoon representation of PVC*pnf* is also depicted (not to scale). TMP = Tape measure protein; AAA+ = ATPases associated with diverse cellular functions; Pnf = *Photorhabdus* necrosis factor; Cif = cycle-inhibiting factor.

Open reading frames (ORFs) at eCIS loci are named according to their encoded order in the operon. PVC loci consist of structural genes *pvc1−5*, *pvc7−13* and *pvc16*, and accessory genes *pvc6*, *pvc14* and *pvc15* (figure 1) [6]. The more diverse operon payloads are encoded downstream of the conserved eCIS core operon and give name to the operon itself, such as PVC*pnf* [9,10].

The *pvc11*-homologous gene in each operon can be used to classify eCIS phylogeny [3]. The minor clade (lineage I) comprises eCISs from Gram-negative bacteria that largely retain each of the core genes, and includes all of the PVC loci. The major clade (lineage II) is more widely distributed in bacteria and archaea and often lacks structural or accessory homologue components such as the *pvc6*, *pvc10* and *pvc14* homologues [3]. Despite sub-clades Ia and Ib having very similar architecture, the functional differences between these sub-clades have not, to our knowledge, previously been described. These sub-clades may differ functionally, as demonstrated by the presence of some key eCISs: lineage Ia includes the PVC and Afp while lineage Ib includes the metamorphosis-associated contractile (MAC) from *Pseudoalteromonas luteoviolacea* and the T6SS^iv^ from *Amoebophilus asiaticus* [3,27,28].

**Figure 2.**
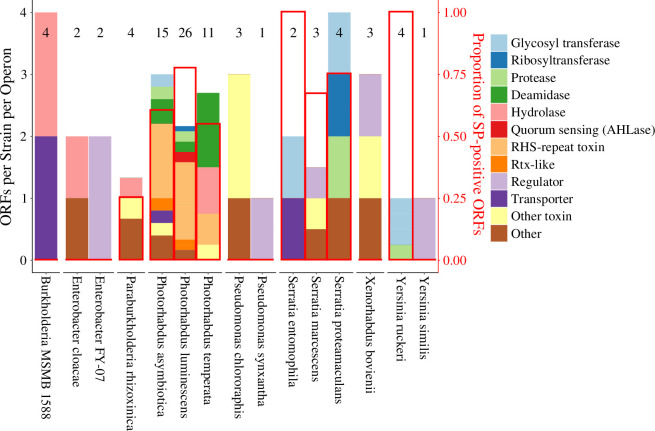
The dbeCIS database was used to identify structural operons of extracellular contractile injection systems (eCIS) in lineage Ia, as defined in [3]. Open reading frames (ORFs) downstream of the final structural gene in the operon were appended to a database where BLASTp and HHPred were used to identify sequence homology and most likely function. Regular expressions were used to count the frequency of keywords using R scripts. ORFs that did not fit any of the assigned keywords were classed as either ‘Other toxin’ or ‘Other’, depending on additional information found for that entry. Numbers presented at the top of each bar represent the total number of ORFs used to generate the stacked bar. Functions were chosen based on the most commonly predicted functions in the database. Genera encoding only one eCIS operon with one or less downstream ORFs were excluded from analysis: lineage Ia *– Chania* and *Shewanella*. AlphaFold2 predictions of each amino acid sequence were made in order to observe whether low-confidence N-terminal signal peptides (SPs) were present. The abundance of these sequences is displayed on the right axis with bars shown as red borders. ‘Protease’ and ‘Nuclease’ are treated as subsets of ‘Hydrolase’ and are preferred to the more ambiguous ‘Hydrolase’ term.

**Figure 3.**
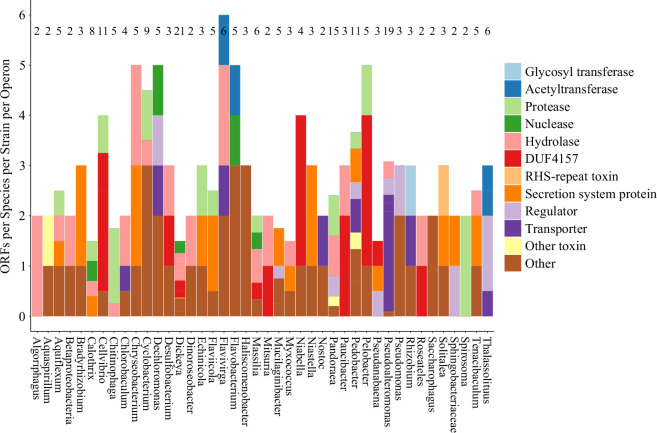
The dbeCIS database was used to identify structural operons of extracellular contractile injection system (eCIS) in lineage Ib, as defined in [3]. Open reading frames (ORFs) downstream of the final structural gene in the operon were appended to a database where BLASTp and HHPred were used to identify sequence homology and most likely function. Regular expressions were used to count the frequency of keywords using R scripts. ORFs that did not fit any of the assigned keywords were classed as either ‘Other toxin’ or ‘Other’, depending on additional information found for that entry. Numbers presented at the top of each bar represent the total number of ORFs used to generate the stacked bar. Functions were chosen based on the most commonly predicted functions in the database. Genera encoding only one eCIS operon with one or less downstream ORFs were excluded from analysis: *Gynuella*, *Minicystis*, *Sorangium*, *Moorea*, *Geobacter*, *Dyadobacter*, *Draconibacterium*, *Anabaena*, *Aulosira*, *Tolypothrix*, *Fremyella*, *Herbaspirillum*, *Tenderia* and *Stigmatella*.

The eCIS database (dbeCIS; http://www.mgc.ac.cn/dbeCIS/) was used to analyse the functional diversity of putative effectors [3,29]. The function of ORFs found immediately downstream of eCIS operons was predicted by sequence and domain architecture using BLASTp and HHPred, respectively [30,31]. Regular expressions counted the frequencies of functions and their distributions in lineage Ia (figure 2) and Ib (figure 3).

AlphaFold2 accurately predicted the structures of native effectors from *E. coli*, *Burkholderia pseudomallei* and *Photorhabdus luminescens* with a root mean square deviation (RMSD) below 0.9 Å against the cryo-EM-resolved structures (electronic supplementary material, figure S1) [24,32–34]. Lineage Ia SPs could be inferred by disordered N-terminal extensions (electronic supplementary material, figure S2A,B) [9,10] which possessed a lower predicted local distance difference test (pLDDT) [24]. SP-containing ORFs had amino acids 1–50 possessing an average pLDDT <40, considerably lower than that of the rest of the ORF. SPs were found in 55.4% of lineage Ia eCIS effectors (figure 2).

All-versus-all analysis of SPs indicates rare homology, as previously documented [9]. However, using the per cent identity matrices to generate a heatmap enabled classification into more nuanced groups (electronic supplementary material, figure S3). These SP groups are termed according to some of their notable effectors: Cif-u3, Rtx, Rhs-*Plum*, LopT, Pnf and Tcc-like.

### 3.2. Supersecondary structures and motifs of the Pvc15 AAA domains

**Figure 4.**
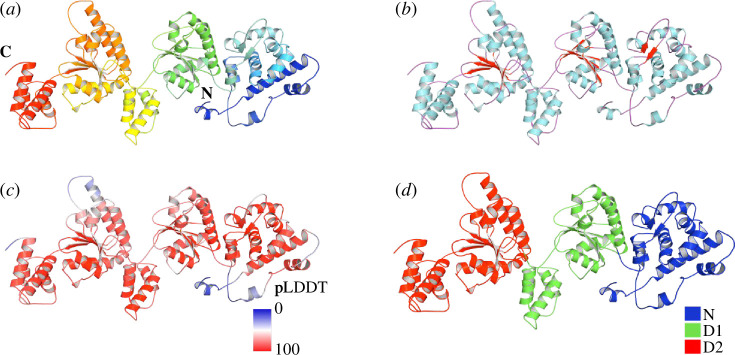
AlphaFold was used to predict the structure of Pvc15*_pnf_* to identify features typical of AAA+ ATPases. Pvc15 is a type II AAA+ ATPase represented by residue number (*a*), secondary structure (*b*), predicted local distance difference test (pLDDT) (0 = lowest score, 100 = highest score) (*c*), and domain architecture (*d*).

The ColabFold suite predicted the structure of Pvc15 encoded at the Pnf operon, hereby denoted as Pvc15*_pnf_* [24,25,35], including amber forcefield relaxation [36]. Pvc15 is a putative AAA+ domain-containing protein homologous to Msm0858 from *Mycolibacterium smegmatis* (5E7P) [37]. Like Msm0858, Pvc15 has an N domain, spanning residues 1–205, and tandem AAA domains D1 (residues 206–434) and D2 (residues 435–698) (figure 4). The pLDDT averaged above 70 in D1 and D2, indicating a confident structure prediction consistent across Pvc15 homologues (electronic supplementary material, figure S4). At the centre of the ATPase domain is a β-α-β sandwich [38], which is a hallmark of AAA+ proteins ordered as β5–β1–β4–β3–β2 [39].

**Figure 5.**
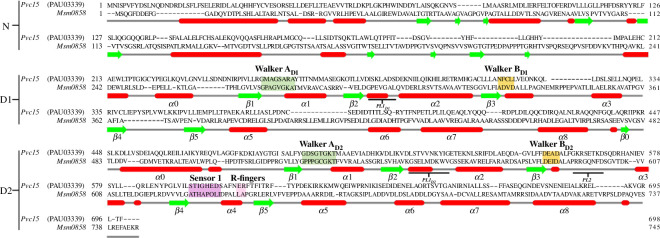
Walker A (green boxes) and B motifs (orange boxes) are conserved ATP binding sites found in Pvc15 homologues including Msm0858 from *Mycobacterium smegmatis* in domains D1 and D2. The second region of homology includes the sensor 1 N-residue (purple box) and arginine fingers (pink box). Helices are indicated by red bars and β-strands are indicated by light green bars.

Alignment with Msm0858 (figure 5) indicates that the Pvc15 domains D1 and D2 are typical of a clade 3 AAA+ ATPase: two arginine fingers (R-fingers) and a short helix following β2 [40]. In D2, the Walker A motif consists of a glycine-rich loop situated between β1 and α1. The ‘GTGK’ motif in Walker A forms a compound nest, which coordinates the β-phosphate of ATP or ADP within the concave surface of the NH bonds, as previously proposed for Ras (electronic supplementary material, figure S5) [41].

**Figure 6.**
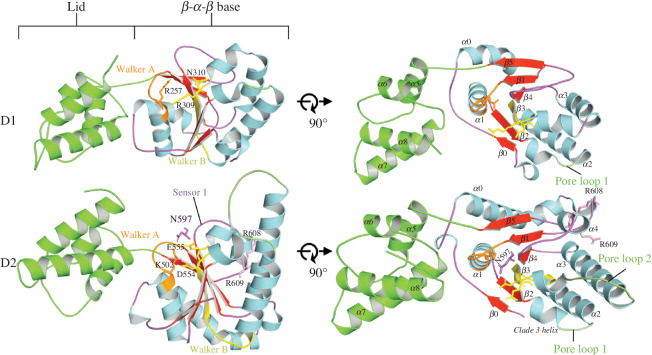
The AlphaFold-predicted structure of Pvc15*_pnf_* was examined to identify key residues of its ATPase domain. The α/β sandwich and lid subdomains of Pvc15 D1 and D2. G499–K502 in the Walker A motif coordinate ATP while D554 and E555 in the Walker B motif catalyse ATP hydrolysis. Sensor 1 residue N597, sensor 2 residue R659 and the more distant Arg finger residues, R608 and R609, are conserved features.

Walker B motifs tend to be less well conserved but often feature up to four hydrophobic amino acids followed by a ‘DE’. In Pvc15, these residues are part of a ‘DEAD’ motif in D2 (figure 6; orange box). The aspartate residue D554 may coordinate a divalent cation such as Mg^2+^, while the glutamate residue E555 is responsible for ATP hydrolysis. Previous work demonstrated that Pvc15 unfolds SP-associated fluorescent proteins, which mutations in Walker A and B motifs failed to abolish [9]. Thus, it was hypothesized that Pvc15 may have other roles outside of its ATPase activity.

### 3.3. Pvc15 hexamers are asymmetrical with pore loops for substrate translocation

MoLPC was used to predict the most likely complete hexameric assembly of Pvc15 (electronic supplementary material, figure S6) [26]. The asymmetrical structure has a pore diameter of 13.7 Å at the D2 domains (electronic supplementary material, figure S6A) and an interface pLDDT (pLDDT_IF_) of 61, indicating good confidence. N-terminal domains assume an open conformation. Each subunit neighbours the next at an angle of between 9° and 12°, producing a right-handed ‘spiral staircase’ such that the two subunits at the ‘top’ and ‘bottom’ are adjacent at approximately 46°.

PL1 and PL2 spiral in accordance with their domains and represent two ‘faces’ capable of contact with the translocating substrate (electronic supplementary material, figure S6B). Lys528 and Tyr529 likely form a cation–π interaction, which suggests a strong binding to substrate, while Arg657 may contact negatively charged amino acids in the substrate [42].

Finally, R-fingers Arg608 and Arg609 are within a Walker B-adjacent cleft in the hexameric structure, which would enable them to stabilize the negative charge of the γ phosphate during ATP hydrolysis *in trans* (electronic supplementary material, figure S6C).

### 3.4. Signal peptides have differential effects on cargo stability

Pvc15 is necessary for loading of cargo proteins to the PVC [9]. To establish whether Pvc15 stabilizes the cargo and improves PVC loading efficiency, SP-tagged cargo abundance was investigated in cell lysates.

BL21(DE3) cells were transformed with plasmids encoding PVC*pnf* operon genes *pvc1−16* (pBAD-PVC*pnf*) and a plasmid encoding the SP-tagged payload. The full 50 amino acid Pnf SP, Pnf50, was linked via a short ‘VD’ (MLKYANPQTVAKAQRTKNTAKKPPSSTSFDGHLELSNGENQPYEGHKIRKIVD) linker to Pnf residues 51–340 and a C-terminal Myc epitope tag.

**Figure 7.**
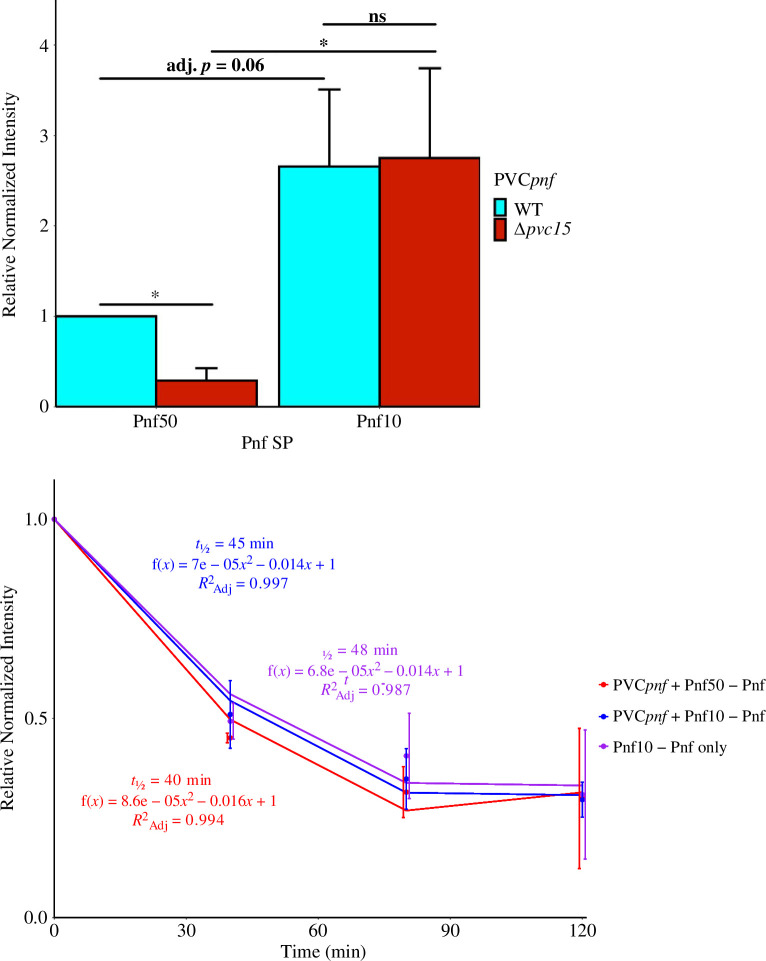
Western blot was used to measure the abundance of Pnf signal peptide-tagged Pnf in *E. coli* lysates. Linear regressions were used to measure its decay over time after the addition of 20 µg ml^−1^ kanamycin. Quantified bands were normalized by the sum of each lane in a Coomassie blue stain. Error bars indicate standard deviation of three biological replicates (*n* = 3); *p < 0.05, ns = not significant.

Western blot was used to measure the effects of SP length and Pvc15 presence on payload abundance and stability in *E. coli* cell lysates. SP-Pnf-Myc was co-expressed in the presence (PVC*pnf*) or absence of Pvc15 (PVC*pnf*Δ*pvc15*) and lysate aliquots were collected at 3 h post-induction (figure 7; electronic supplementary material, figure S7(i)). Pnf band intensities showed a decrease in the stability of Pnf50-Pnf-Myc when Pvc15 was removed (*t*(4) = −5.55, *p* = 0.005, adj. *p* = 0.02*), indicating that Pvc15 acts to increase cargo abundance in the lysate. Truncation of the 50 amino acid SP by 40 amino acids from its C-terminus (Pnf10: MLKYANPQTVVD) made Pnf more stable than when it was encoded with the native Pnf50 SP, regardless of whether Pvc15 was present (*t*(4) = 3.30, *p* = 0.030, adj. *p* = 0.06) or absent (*t*(4) = 4.26, *p* = 0.013, adj. *p* = 0.039*). This suggested that Pnf50 has a destabilizing effect on the cargo, which is mitigated when Pvc15 is co-expressed.

The addition of kanamycin to block protein synthesis indicated that the effects on protein abundance were due to differences in protein degradation rather than translation (figure 7; electronic supplementary material, figure S7(ii,iii)). Half-lives ranged from 40 to 48 min using a quadratic equation of decay and all samples had similar coefficients. Altogether, this indicates that Pnf alone is rapidly degraded but rescued by the co-expression of Pvc15.

**Figure 8.**
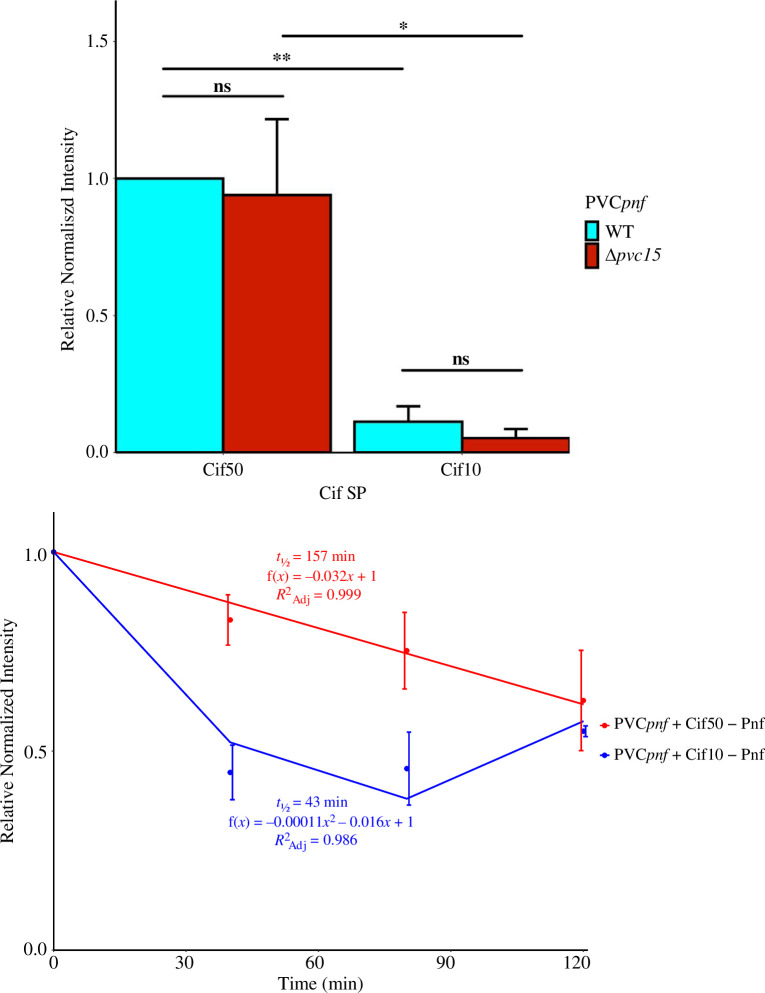
Western blot to measure the abundance of Cif SP-tagged Pnf in *Escherichia coli* lysates and linear regressions to measure its decay over time after the addition of 20 µg ml^−1^ kanamycin. Quantified bands were normalized by the sum of each lane in a Coomassie blue stain or by the loading control, β-RNAP. Error bars indicate standard deviation of 2 or 3 biological replicates (*n* = 2 or 3); *p < 0.05, **p < 0.01, ns = not significant.

To test whether other SPs have a similarly destabilizing effect without Pvc15*_pnf_*, the Cif SP (Cif50) with ‘VD’ linker (Cif50; MREYSKEDDCVKEKTNLAESENVEADNYLEMDCLNYLAKLNGMPERKDHSVD) was encoded in place of the Pnf50 SP N-terminal of the Pnf cargo. Pvc15 had no effect on the stability of Cif50-Pnf-Myc nor the similarly C-terminal-truncated Cif SP (Cif10; MREYSKEDDCVD), Cif10-Pnf-Myc, though the latter was less stable compared with the longer SP both with and without Pvc15 (*t*(3) = −11.28, *p* = 0.0013, adj. *p* = 0.0052**; *t*(4) = −5.51, *p* = 0.005, adj. *p* = 0.016*) (figure 8; electronic supplementary material, figure S8(i–iii)). For the Cif SP, differences in abundance were explained by differences in stability since Cif50-Pnf-Myc was almost fourfold more stable than Cif10-Pnf-Myc. Thus, Pvc15*_pnf_* interacts differentially between the different PVC cargo SPs, and SPs differentially alter a payload’s innate stability.

### 3.5. Signal peptides are selected by Pvc15 for *Photorhabdus* virulence cassette loading

**Figure 9.**
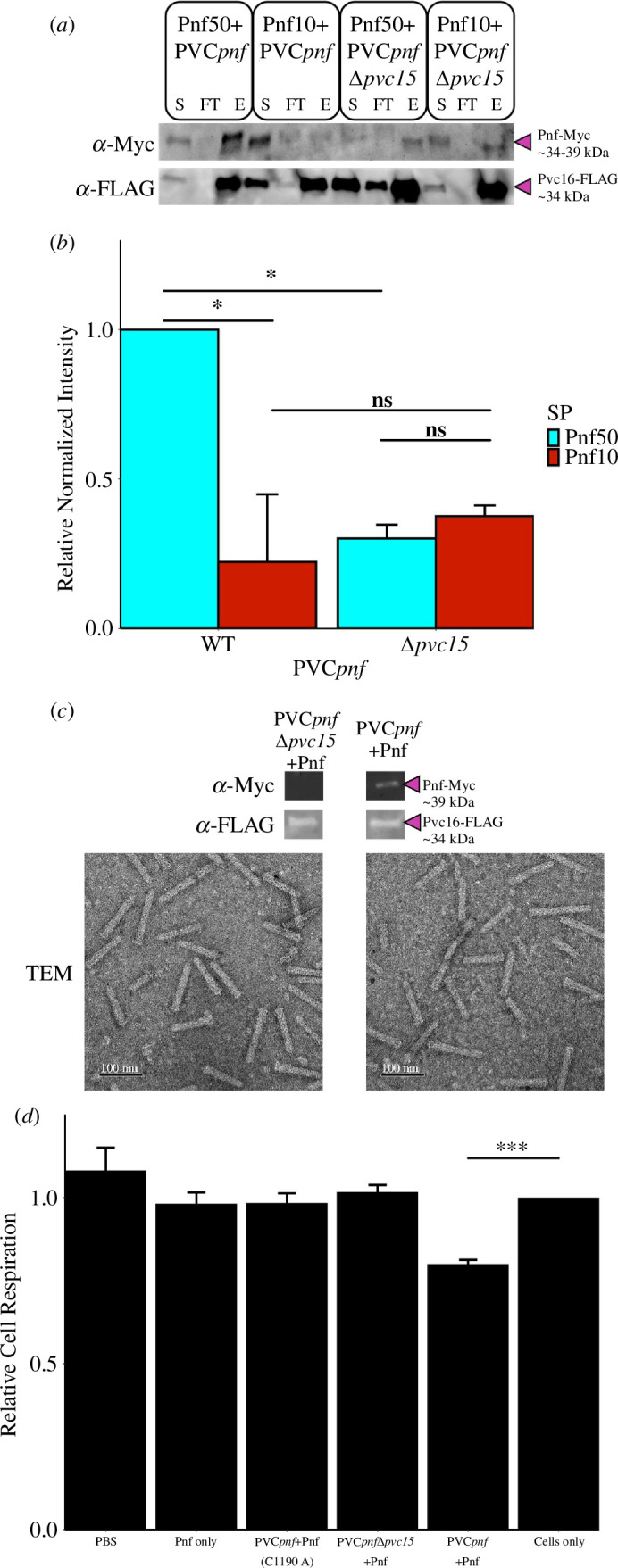
(*a,b*) Western blot of Pnf cargo co-purification with PVC*pnf*, supernatant (S) and flow-through (FT). Elutions (E) are quantified and normalized by Pvc16-FLAG (*n* = 2 or 3 reiterations of purified PVC*pnf*; two-sided Student’s *t*‐test; *p < 0.05, ns = not significant). (*c*) PVC*pnf* and PVC*pnf*Δ*pvc15* were further purified and assayed by western blot and transmission electron microscopy (TEM). (*d*) The effects of PVCs on relative resazurin reduction by Raji cells were then used as a measure of cell respiratory activity and viability (*n* = 4; two-sided Student’s *t*‐test; ***p < 0.005. Data are representative of Raji cells, though similar results were found for Jurkat T cells and S2 macrophage-like *Drosophila* cells (electronic supplementary material, figure S10). Error bars show standard deviation.

To compare loading efficiencies into PVC*pnf*, nanosyringes were purified using co-immunoprecipitation of an epitope-tagged PVC protein. Western blot was used to quantitatively measure payload abundance relative to Pvc16-FLAG in eluted samples (figure 9*a*, lanes ‘E’) (electronic supplementary material, figure S9 and additional replicates in electronic supplementary material, figure S15). Loading efficiencies were 50–75% lower for cargoes with truncated Pnf10 (*t*(3) = −3.22, *p* = 0.049*) and those co-expressed in the absence of Pvc15 (*t*(4) = −4.23, *p* = 0.013*; figure 9*b*). Background levels of Pnf payload from PVC*pnf*Δ*pvc15* nanosyringes were cleared after further PVC purification (figure 9*c*). As shown in previous studies [6,9,10], PVC*pnf*Δ*pvc15* nanosyringes were intact when imaged in transmission electron microscopy but lacked payload. These results confirm that truncated SPs, while conferring cargo stability, fail to recruit cargo to the PVC at comparable levels.

**Figure 10.**
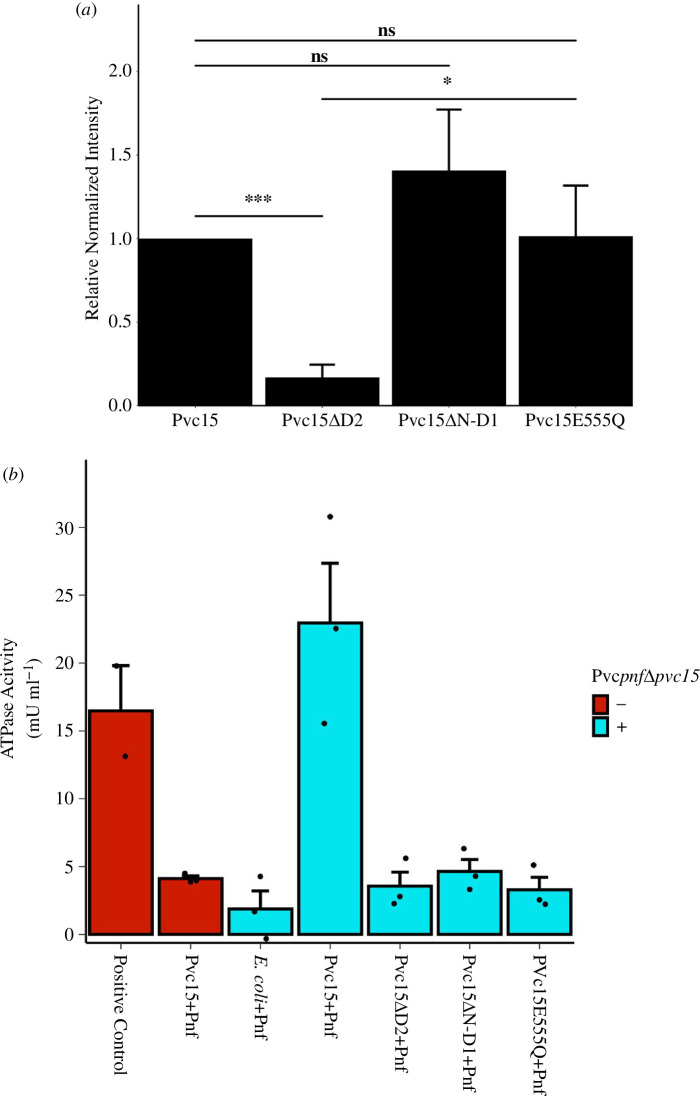
(*a*) *E. coli* lysates expressing Pnf50-Pnf-Myc alongside wild-type (WT) or mutant Pvc15 via the pVTRaDuet vector were analysed by western blot for Pvc15’s ability to stabilize Pnf50-Pnf-Myc. The abundance of Pnf50-Pnf-Myc was normalized by the corresponding abundance of Pvc16-FLAG. Error bars show standard deviation of three biological replicates (*n* = 3). (*b*) A colorimetric ATPase assay was used to confirm whether each of these Pvc15 mutants retained ATPase activity. As a measure of the precision of the measured ATPase activity, error bars indicate standard error of the mean of three biological replicates (*n* = 3). *p < 0.05, ***p < 0.005, ns = not significant.

A resazurin assay showed that Raji B cells, calibrated to an appropriate cell density (electronic supplementary material, S10), demonstrated a decrease in relative respiratory activity when incubated with Pnf-loaded PVC*pnf* (figure 9*d*) (*t*(6) = −9.95, *p* = 5.569 × 10^−5^, adj. *p* = 0.00028***). Pnf with an active site mutation (C190A) [7], as well as PVC*pnf*Δ*pvc15* nanosyringes, did not affect cell respiration. Additionally, Ni^+^ nitrilotriacetic acid (NTA)-purified Pnf payload without PVC*pnf* did not affect cell respiration, indicating the importance of the PVC*pnf* vehicle. Experiments in Jurkat T cells and *Drosophila* S2 cells displayed similar results: PVCs lacking payloads did not affect cell viability (electronic supplementary material, S10).

### 3.6. Vector construction for Pvc15 mutant investigation

A pVTRa vector encoding the native Pnf50-Pnf-Myc downstream of double polyhistidine-tagged Pvc15 was constructed and termed pVTRaDuet (electronic supplementary material, figure S11A). Pnf co-expressed with Pvc15 for 20 h post-induction resulted in loss of detectable cargo (electronic supplementary material, figure S11B), though cargo levels were reconstituted when co-expressed with the rest of the PVC*pnf* operon (electronic supplementary material, figure S11B: lanes 4–6 compared with lanes 10–12). This may suggest that additional interactions mediate cargo stability after overnight PVC*pnf* induction. In addition, PVC*pnf*Δ*pvc15* induction does not affect Pvc15’s ability to stabilize Pnf (electronic supplementary material, figure S12).

### 3.7. Pvc15 domain D2 stabilizes Pnf50 pump’s cargo into PVCpnf using its ATPase

Pvc15 mutants with deletions of the N to D1 domains (Pvc15ΔN-D1), the D2 domain (Pvc15ΔD2) or mutation of the catalytic E555 (Pvc15E555Q) were constructed. pBAD-PVC*pnf*Δ*pvc15* was co-expressed along with Pnf50-Pnf-Myc for 3 h. Aliquots were analysed by western blot and normalized by cellular β-RNA polymerase (β-RNAP: approx. 150 kDa; figure 10*a*; electronic supplementary material, figure S13).

Deletion of Pvc15 D2 depleted Pnf50-Pnf-Myc in cell lysates (*t*(4) = −8.66, *p* = 0.0010, adj. *p* = 0.0040***), suggesting that this domain is the primary chaperone for SP-tagged cargoes in the bacterium (figure 10*a*). The E555Q mutation, hypothesized to disrupt ATPase activity, did not abolish Pnf abundance (*t*(4) = 0.68, *p* = 0.95). Moreover, deletion of domains N and D1 did not affect the abundance of Pnf (*t*(4) = 1.78, *p* = 0.15, adj. *p* = 0.29), further validating that the D2 domain alone is sufficient for Pnf chaperoning. In fact, compared with the Pvc15ΔD2 mutant, reconstituting with an active site mutant D2 domain, Pvc15E555Q, was sufficient to rescue Pnf abundance (*t*(4) = −4.68, *p* = 0.0094, adj. *p* = 0.028*).

A malachite green ATPase assay was used to measure a change in OD_650_ (figure 10*b*) and normalize to a standard curve (electronic supplementary material, figure S14). In the presence of *pvc1−14* and *pvc16* (PVC*pnf*Δ*pvc15*^+^; cyan), each of the Pvc15 mutants had activity comparable to the negative control (1.9 mU ml^−1^), while wild-type (WT) Pvc15 had greater ATPase activity (23.0 mU ml^−1^), similar to that of the positive control (*t*(3) = −1.05, *p* = 0.37 (n.s.), 95% CI [−20.86, 7.89]). Comparing with the Pvc15+Pnf PVC*pnf*Δ*pvc15*^+^ strain: *E. coli*+Pnf: *t*(4) = −4.59, *p*‐value = 0.010*, 95% CI [−33.85, −8.32]; Pvc15ΔD2: *t*(4) = −4.29, *p*‐value = 0.013*, 95% CI [−31.95, −6.85]; Pvc15ΔN-D1: *t*(4) = −4.08, *p* = 0.015*, 95% CI [−30.78, −5.85]; Pvc15E555Q: *t*(4) = −4.37, *p* = 0.012*, 95% CI [−32.14, −7.18]. Given that mutation of E555, situated within D2, abolishes the ATPase, D2 is likely the sole functional ATPase domain. In addition, ATPase activity was abolished in lysates not co-expressing PVC*pnf*Δ*pvc15* (*t*(4) = −3.18, *p* = 0.033*, 95% CI [−31.07, −6.61]), suggesting that Pvc15’s ATPase activity requires interactions with other PVC*pnf* proteins or even the mature PVC. While the Holm-adjusted *p*-values for each of these significant comparisons was 0.061, the 95% confidence intervals of the non-adjusted *p*-values did not include 0, and the very large effect size (Cohen’s *d* > 3.3) observed between the WT (PVC*pnf*Δ*pvc15* + [Pvc15 + Pnf]) and each of the mutants indicates that these data are of practical significance and warrant further investigation.

**Figure 11.**
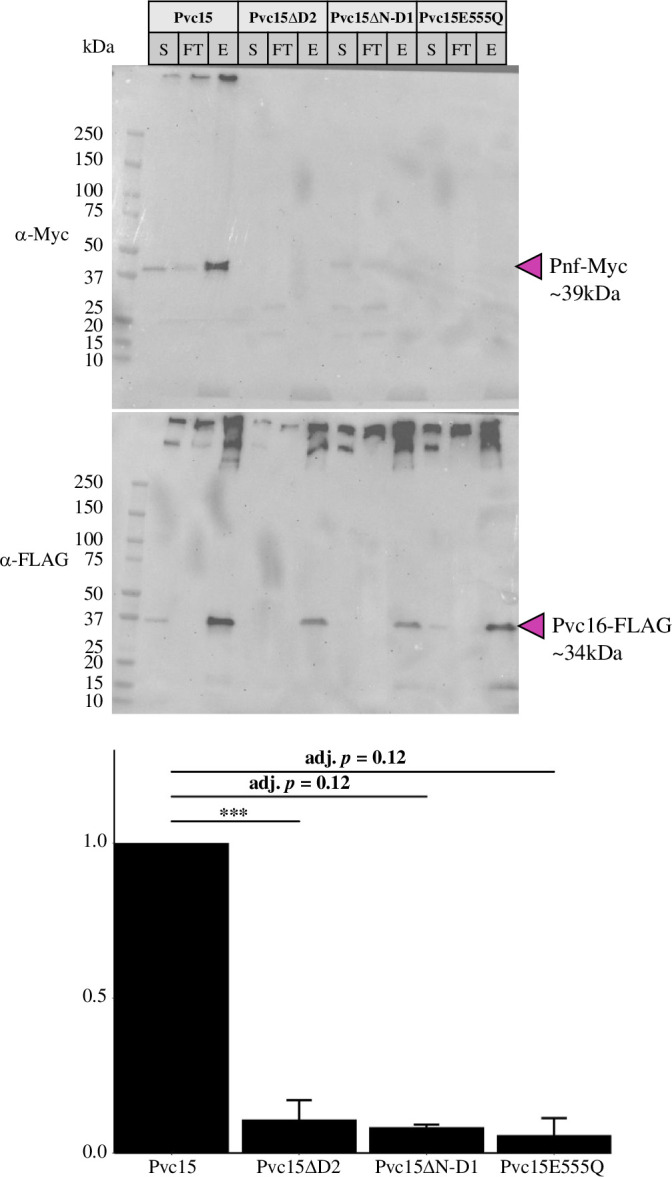
Western blot using α-Myc, α-FLAG and α-β-RNAP primary antibodies against purified PVC*pnf* with co-expression of Pvc15 mutants for quantification of co-purified Pnf50-Pnf-Myc. Normalization was done using the associated Pvc16-FLAG bands. S = supernatant, FT = flow-through, E = elution (purified PVC*pnf*). Data are representative of three reiterations of PVC*pnf* purifications (*n* = 3); ***p < 0.005.

Strains co-expressing ATPase-incompetent Pvc15 mutants showed a lack of associated Pnf payload compared with WT, indicating that ATPase activity was required for effective cargo loading (figure 11; electronic supplementary material, figure S15). Compared with WT Pvc15: Pvc15ΔD2: *t*(3) = −6.01, *p* = 9.2 × 10^−4^, adj. *p* = 0.0037***; Pvc15ΔN-D1: *t*(2) = −4.84, *p* = 0.040, adj. *p* = 0.12; Pvc15E555Q: *t*(2) = −4.88, *p* = 0.039, adj. *p* = 0.12. On the other hand, Pnf appeared to be depleted in the supernatant of the Pvc15 mutant samples. Therefore, our data may suggest that Pvc15 ATPase activity, carried out by E555 in domain D2, is required to translocate payload into the tube lumen of the mature PVC; further work should be carried out to support this hypothesis.

## 4. Discussion

Effector function analysis presented in this study demonstrates that *Photorhabdus* putative effectors are significantly more highly represented in their use of rearrangement hotspot (Rhs) repeat-containing proteins, particularly in *Photorhabdus asymbiotica* and *P. luminescens*, which have been associated with rare cases of human disease [43,44]. On the other hand, eCIS-downstream ORFs of *Photorhabdus temperata* were more commonly predicted to have deamidase and hydrolase activities. This study also found that SPs in lineage Ia are localized almost entirely to the PVC loci in *Photorhabdus* and the Afp loci in *Serratia* and *Yersinia*, implicating disordered SPs as an Afp-homologue-specific feature of putative eCIS cargoes.

Across the 41 lineage Ia core eCIS operons analysed, there are 88 putative effector ORFs, while in the 110 lineage Ib core eCIS operons analysed, there are 240 putative effector ORFs. Twenty-four ORFs across 13 species in lineage Ib (10% of lineage Ib ORFs) had similar architecture to a conserved domain of unknown function (DUF), DUF4157: a putative metallopeptidase. It contains a Zn-binding motif, HexxH, typical of metallopeptidases; authors have speculated its function to be involved in toxin interactions such as for loading or export [22]—a hypothesis that may be reflected in the current work since every instance of DUF4157 was encoded adjacent to another eCIS-downstream ORF with the only exception being *Stigmatella aurantiaca* strain DW4/3-1.

Pvc15’s predicted structure demonstrated it is a classic AAA ATPase, of which the second region of homology (SRH) is a defining characteristic (figure 5). The sensor 1 motif (purple box) residue Asn597 is responsible for the orientation of the water used in hydrolysis. The nearby Arg608 and Arg609 R-fingers (pink box) coordinate with adjacent subunits in the hexameric complex, while pore loops 1 and 2 (PL1 and PL2) likely bind substrate peptide [39].

In Msm0858, both the D1 and D2 domains are capable of ATP hydrolysis [37]. However, Pvc15 D1 has deviant residues in the key catalytic sites compared with D2 (figure 6). In particular, (i) Pvc15 D1 lacks an Asp or Glu residue in the Walker B box, (ii) the Walker A nest Lys is replaced with Arg, and (iii) D1 lacks SRH residues. Altogether, these data may indicate that D1 is deficient for ATPase activity, reminiscent of other AAA+ ATPases such as p97/VCP, whose D1 has minimal *in vitro* ATPase activity but is responsible for stabilizing the hexamer, and NSF, where D1 is an ATPase and D2 is responsible for hexamerization [45–47].

While protein structure analysis revealed potential functions of D1 and D2 Pvc15 domains, the N domain’s function is harder to derive. A search of the DALI server [48] using the Pvc15 N domain indicates many hits for DNA-directed transcription factor subunits, including the RNA polymerase II transcription factor B from *Saccharomyces cerevisiae* with a *Z*-score of 7.5 at 2.9 Å r.m.s.d (PDB: 5OQJ) [49] and also includes the MepR transcriptional regulator from *Saccharomyces aureus* with a *Z*-score of 6.8 and 3.5 Å RMSD over 90 aligned residues (PDB: 4LD5) [50]. Whether the N domain is responsible for nucleic acid binding has yet to be demonstrated; the N domain’s functional role should be investigated in future studies. In addition, the hexamer assembly of Pvc15 may change drastically once bound to substrate and nucleotide since many ATPases, such as Lon, assume a more closed structure during substrate translocation [51]. The mechanodynamics of substrate translocation of Pvc15 should be investigated.

Pvc15 was also found to rescue Pnf50-Pnf from rapid degradation (figure 7). Of note, strains encoding—but actively repressing—Pvc15 still rescued Pnf50-Pnf, suggesting that Pvc15 exerts a chaperoning effect even at low cytosolic levels through leakage from the uninduced plasmid. Given the interpretation of Pvc15’s binding of Pnf50, Pvc15 is predicted to rescue 30% of cytosolic Pnf from degradation (95% CI [15%, 43%]), calculated by comparing with the abundance of the more stable Pnf10. Investigation of how other Pvc15 homologues, such as Pvc15*_cif_*, might influence different SPs would be insightful for future studies. Such investigations would allude to whether PVC chassis are promiscuous in their loading of multiple effectors simultaneously, or whether each PVC locus is intended for only loading a subset of cargoes. The latter ‘PVC specificity’ model may be suspected given that each of the *P. luminescens* PVC operons is thought to have arisen from horizontal gene transfer [7]; if true, then the specific interaction ‘keys’ responsible for ‘unlocking’ the PVC chassis may belong to the SP associated with the effectors, the Pvc15 associated with the operon, or both.

As shown by recent data, PVCs can be artificially targeted to certain cell receptors to ensure optimal payload injection and cell type specificity whereby Pnf is cytotoxic to the target cells [10]. Pnf acts similarly to *E. coli* cytotoxic necrotizing factor, by deamidating a crucial residue in RhoA, which subsequently causes the formation and maintenance of stress fibres [7,33]. Furthermore, cytoskeletal disruption interferes with mitochondrial activity via voltage-dependent anion channels and mitochondrial creatine kinase [52]. Thus, resazurin can be used to measure the fluorescence of the mitochondria-reduced form, resorufin, as a measure of cell viability in response to Pnf injection by PVC*pnf* (figure 9*d*). For Raji B cells, the optimal starting cell concentration for a 24 h time course was found to be 8 × 10^5^ cells ml^−1^ (electronic supplementary material, figure S8).

The results presented in this study found that the Pnf SP is necessary for payload loading, though it does not require cleavage to be loaded into PVC*pnf* since purified samples resulted in Pnf molecular weights corresponding to uncleaved SPs (approx. 39 kDa; electronic supplementary material, figure S7). Smaller bands observed in *E. coli* lysates (approx. 25 and 17 kDa) are also present when expressing Pnf. Thus, these lower bands may be the result of a consistent Pnf degradation pattern by the cell rather than specific cleavage of the SP for loading.

Pnf-loaded PVC*pnf* was found to affect mammalian immune cell respiration. Therefore, while wild-type tail fibres are not expected to bind promiscuously [10], some background binding to non-target cells could be sufficient to cause effects on cell viability when injecting cytotoxic cargo. This is notable for the future design of PVCs as biotherapeutic delivery agents since some off-target injections may take place upon eukaryotic cell membranes.

## 5. Conclusion

This study showed that each of Pvc15’s domains has a crucial role in the chaperoning and association of SP-associated cargo proteins with the PVC via the ATPase activity. SPs are ubiquitous in other homologous eCIS putative cargoes such as the Afp in *Serratia* and *Yersinia*. Despite their similar role in PVC maturation, SPs act differentially with Pvc15; their groups of homologies between similar strains and operons in *Photorhabdus* may give clues as to whether certain conserved motifs within SPs have similar roles or protein-protein interactions.

The Pvc15 N domain was found to have homology to DNA-binding proteins, while the D1 domain may be involved in stabilizing the hexamer. The D2 domain plays a more significant role in ATPase activity, SP stabilization and possibly PVC loading. In this study, Pvc15 ATPase activity was demonstrated to mediate the loading of SP-tagged payloads into the PVC after stabilizing them in the bacterial cytosol. Despite previous works showing Pvc15’s ability to unfold tagged cargoes prior to loading, injected payloads still exert the effects of the refolded protein. Understanding the functional roles of the SP, Pvc15 and interactions with other PVC components will enable researchers to facilitate the potential use of these devices for eukaryotic injection of bioactive therapeutic proteins in the future.

## Data Availability

The data supporting the findings of the article are available in the Zenodo repository at [53]. Supplementary material is available online [54].
